# Meta-analysis of Cholesteryl Ester Transfer Protein TaqIB Polymorphism and Risk of Myocardial Infarction

**DOI:** 10.1097/MD.0000000000000160

**Published:** 2014-12-05

**Authors:** Min Cao, Zhi-Wen Zhou, Bang-Jiang Fang, Cheng-Gen Zhao, Duan Zhou

**Affiliations:** From the Department of Emergency, Longhua Hospital Afflicted to Shanghai University of Traditional Chinese Medcine (MC, J-BF); Department of Cardiology, Xuhui District Central Hospital, Shanghai, China (Z-W Z); Department of Traditional Chinese Medicine, Putuo Hospital Afflicted to Shanghai University of Traditional Chinese Medcine (G-GZ); and Department of Cardiology, Longhua Hospital Afflicted to Shanghai University of Traditional Chinese Medcine, Shanghai (DZ), China.

## Abstract

A number of studies have been conducted to explore the association between the cholesteryl ester transfer protein (CETP) TaqIB polymorphism and risk of myocardial infarction (MI); however, the results are inconsistent. Therefore, we conducted this meta-analysis to clarify the issue based on all the data available.

Eligible studies were retrieved by searching PubMed, Embase, Web of Science, and Google Scholar. We calculated the crude odds ratios (ORs) and corresponding 95% confidence intervals (95% CIs) to assess the association between the TaqIB polymorphism and risk of MI.

We included 13 studies involving 8733 MI cases and 8573 controls in the meta-analysis. The pooled results from all included studies showed decreased MI risk in the analysis of the B2B2 versus B1B1 (OR = 0.78, 95% CI = 0.68–0.91), dominant (OR = 0.88, 95% CI = 0.77–0.99), and recessive genetic models (OR = 0.84, 95% CI = 0.78–0.91). The frequency of the B2B2 genotype in MI patients was lower (OR = 0.87, 95% CI = 0.81–0.94). However, there was no significant association in the B1B2 versus B1B1 analysis (OR = 0.92, 95% CI = 0.81–1.05) and no significant difference for the B1B1 genotype (OR = 1.04, 95% CI = 0.98–1.11) and B1B2 genotype (OR = 1.03, 95% CI = 0.97–1.08). Cumulative analysis confirmed these results.

Our results suggest that the B2B2 genotype of the *CETP* TaqIB polymorphism is a protective factor against the development of MI.

## Abstract 2

Supplemental Digital Content is available in the text

This work was supported by grants from the National Natural Science Foundation of China (No. 81202660), and the 2013 Overseas Study Plan of Middle-aged and Young College Teachers in Shanghai and the Traditional Chinese Nova Program of Shanghai (ZYSNXD011 -RC- XLXX-20130001). The funders had no role in study design, data collection and analysis, decision to publish, or preparation of the manuscript.

## INTRODUCTION

Myocardial infarction (MI) is one of the leading causes of death in humans, and is a complex disease influenced by modifiable risk factors as well as genetic susceptibility.^1^ It has been reported that the heritability of MI ranges between 25% and 60%.^2,3^ In fact, other than the traditional risk factors, such as smoking, obesity, hypertension, dyslipidemia, and diabetes, numerous studies have revealed the importance of genetic factors in the pathogenesis of MI.^4–6^

It is well known that abnormal plasma lipid and lipoprotein metabolism is an independent risk factor for MI, and is closely related to genetic factors.^7^ Cholesteryl ester transfer protein (CETP) mediates the transfer of cholesteryl esters and triglycerides from high-density lipoprotein cholesterol (HDL-C) to low-density lipoprotein cholesterol (LDL-C) and to very-low-density lipoprotein (VLDL) cholesterol, thus playing a crucial role in reverse cholesterol transport.^8^ CETP dysfunction causes alterations in plasma lipids and therefore contributes to the occurrence of MI.^9,10^ Given its unique physiological role in reverse cholesterol transport, *CETP* is considered as an interesting candidate gene for studying susceptibility to coronary heart disease (CHD) and MI.

The *CETP* gene is located on 16q12–21 and contains 16 exons and 15 introns encoding 476 amino acids. Many single-nucleotide polymorphisms have been found in this gene, the most extensively studied of which is TaqIB (also named rs708272), located in nucleotide 277 of intron 1.^11^ The mutation in this position is recognized by the *Taq*I restriction enzyme, and it forms 3 genotypes: B1B1, B1B2, and B2B2. It has been reported that the TaqIB polymorphism influences the concentration and activity of plasma CETP, apolipoprotein A1 (apoA-I), and HDL levels,^12–14^ and may contribute to the pathogenesis of coronary artery disease (CAD) or MI. In fact, some meta-analyses have provided evidence that the TaqIB polymorphism is significantly associated with risk of CAD in B2B2 individuals as compared with B1B1 individuals.^15–17^ A number of studies have assessed the association between the TaqIB polymorphism and risk of MI^18–30^; however, the results have been inconsistent. A recently published meta-analysis^31^ explored the association between the TaqIB polymorphism and risk of MI; however, it included only 5 case–control studies, missing many other types of studies; consequently, its conclusion may not be reliable. Therefore, we conducted this meta-analysis to draw a reasonable conclusion regarding the association between the *CETP* TaqIB polymorphism and risk of MI.

## METHODS

### Search Strategy

Eligible articles were retrieved by searching PubMed, Embase, Web of Science, and Google Scholar (up to April 16, 2014) using the following keyword combinations: CETP OR cholesteryl ester transfer protein OR TaqIB OR rs708272; acute coronary syndrome OR myocardial infarction; polymorphism OR polymorphisms OR variants OR variant. In addition, we checked the references in the retrieved articles to identify other potential articles. There were no language restrictions.

### Inclusion and Exclusion Criteria

The inclusion criteria were: full-text articles on the relationship between the TaqIB polymorphism and MI risk and sufficient data for estimating an odds ratio (OR) with 95% confidence interval (CI). We excluded studies that contained no usable data, that were systematic reviews, or that were unrelated to MI or the TaqIB polymorphism.

### Data Extraction

Two of the authors extracted the relevant data from all included studies using a predesigned data extraction table. The following information was extracted: first author, year of publication, ethnicity and country involved, sample size, genotype frequencies, and evidence of Hardy–Weinberg equilibrium (HWE).

### Statistical Analysis

We used STATA statistical software (version 11; StataCorp, TX) for the statistical analysis. The crude ORs and corresponding 95% CIs were calculated to assess the association between the TaqIB polymorphism and risk of MI for the following 4 genetic models: B2B2 versus B1B1 (B2, minor allele; B1, major allele); B1B2 versus B1B1; dominant (B2B2 + B1B2 vs B1B1); and recessive (B2B2 vs B1B2 + B1B1). The frequencies of the B1B1, B1B2, and B2B2 genotype were also calculated using the same method. We also performed cumulative meta-analysis for the above genetic models. HWE was tested using a chi-square (χ^2^) test in the control populations. We evaluated potential heterogeneity between studies using a *χ*^2^ test and the *I*^2^ statistic. A fixed effects model was used if there was no heterogeneity; otherwise, we used a random effects model. Sensitivity analysis was performed to assess the influence of single studies on the overall ORs. Potential publication bias was calculated using Begg and Egger tests. A *P* value of <0.05 was considered statistically significant.

## RESULTS

### Study Selection and Characteristics of Included Studies

**FIGURE 1:**
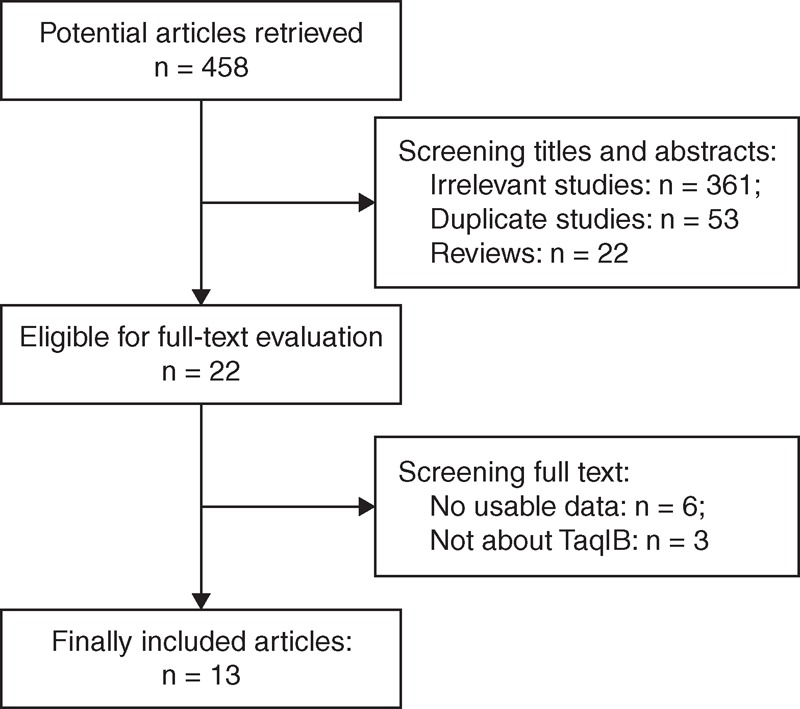
Flowchart of the study selection process.

We retrieved 458 studies from PubMed, Embase, Web of Science, and Google Scholar, and excluded 436 after reviewing their titles and abstracts (361 irrelevant studies, 53 duplicate studies, 22 reviews); 22 full texts were evaluated, of which 9 were excluded (6 with no usable data, 3 were unrelated to the TaqIB polymorphism). We eventually included 13 studies involving 8733 MI cases and 8573 controls in our meta-analysis. The detailed selection procedure is depicted in Figure 1. In the studies of Wu et al,^22^ Keavney et al,^25^ and Thomas et al,^28^ the genotype distributions of the controls were not in HWE. Table 1 details the characteristics of the studies. The present study met the PRISMA statement requirements (Table S1, http://links.lww.com/MD/A120 and Figure 1).

**TABLE 1:**
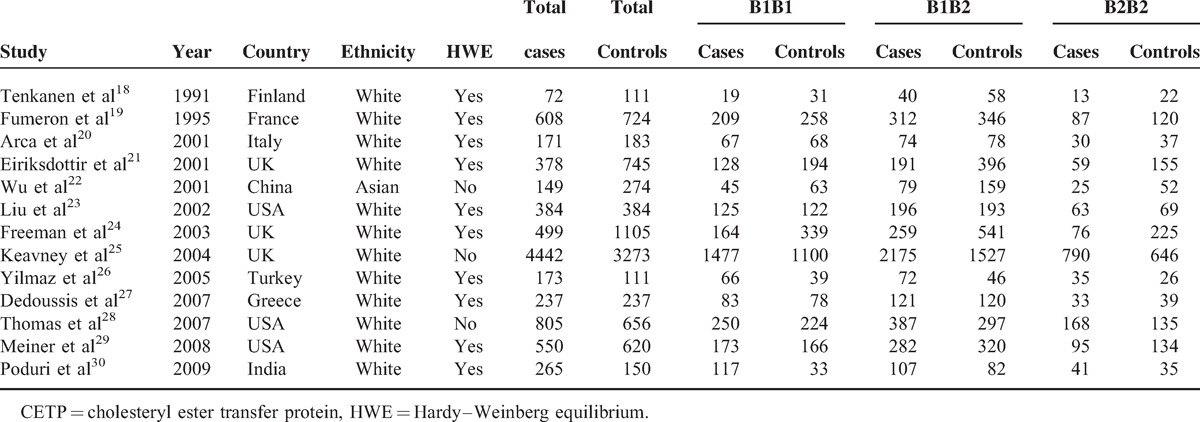
Characteristics of Studies Included in CETP TaqIB Polymorphisms and Myocardial Infarction

### Quantitative Data Synthesis

**FIGURE 2:**
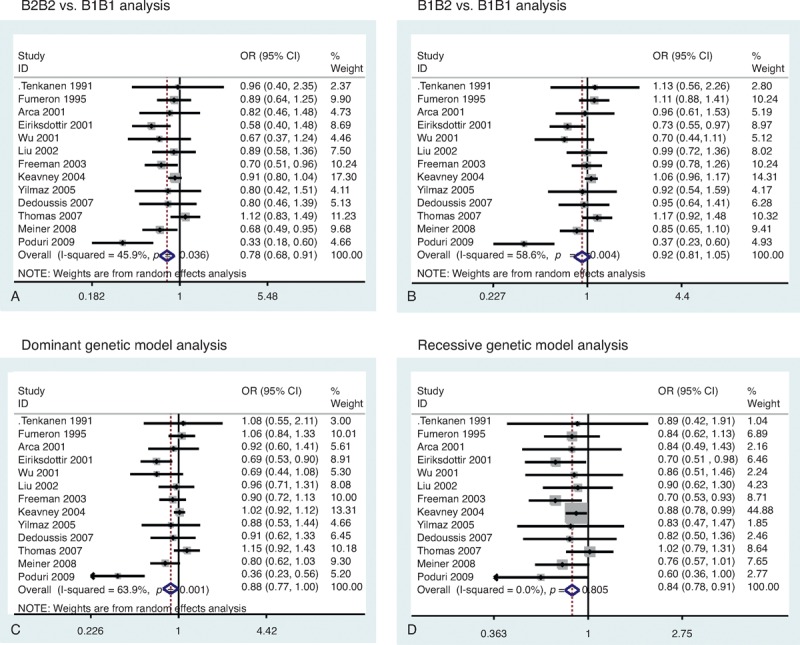
Funnel plot of *CETP* TaqIB polymorphism and MI risk. (A) B2B2 vs B1B1 analysis. (B) B1B2 vs B1B1 analysis. (C) Dominant genetic model analysis. (D) Recessive genetic model analysis. CI = confidence interval, MI = myocardial infarction, OR = odds ratio.

**FIGURE 3:**
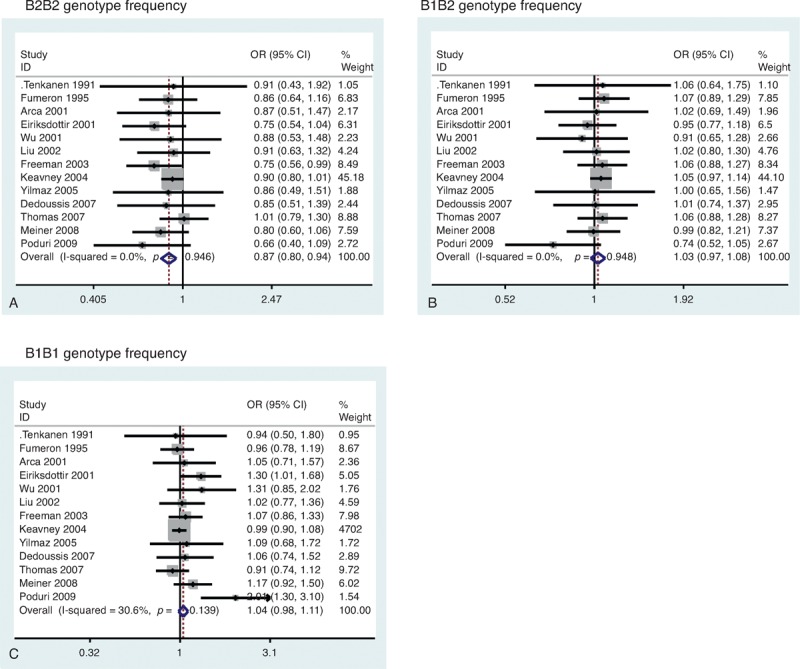
Funnel plot of *CETP* TaqIB polymorphism and MI risk. (A) B2B2 genotype frequency. (B) B1B2 genotype frequency. (C) B1B1 genotype frequency. CI = confidence interval, MI = myocardial infarction, OR = odds ratio.

**FIGURE 4:**
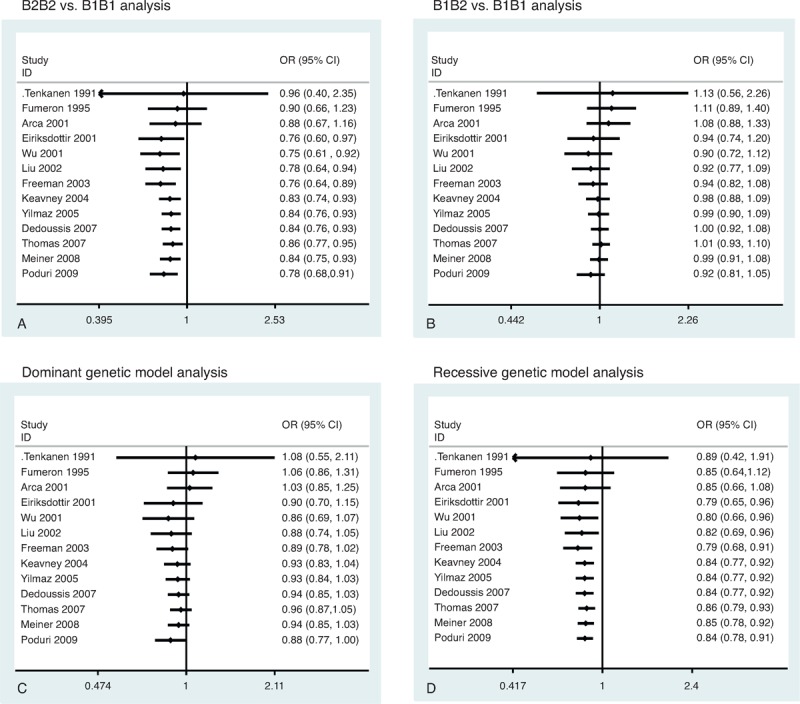
Cumulative analysis of *CETP* TaqIB polymorphism and MI risk. (A) B2B2 vs B1B1 analysis. (B) B1B2 vs B1B1 analysis. (C) Dominant genetic model analysis. (D) Recessive genetic model analysis. CI = confidence interval, MI = myocardial infarction, OR = odds ratio.

Our meta-analysis showed that there was a significant association between the TaqIB polymorphism and risk of MI. Significantly decreased MI risk was determined from the analysis of the B2B2 versus B1B1 (OR = 0.78, 95% CI = 0.68–0.91, *P* = 0.001) (Figure 2A), dominant (OR = 0.88, 95% CI = 0.77–0.99, *P* = 0.045) (Fig. 2C), and recessive genetic models (OR = 0.84, 95% CI = 0.78–0.91, *P* < 0.001) (Fig. 2D). However, the B1B2 versus B1B1 analysis (OR = 0.92, 95% CI = 0.81–1.05, *P* = 0.21) (Fig. 2B) revealed no significant associations. There was a lower frequency of the B2B2 genotype in MI patients (OR = 0.87, 95% CI = 0.81–0.94) (Figure 3A). However, there was no significant difference for the B1B1 genotype (OR = 1.04, 95% CI = 0.98–1.11) (Fig. 3B) and B1B2 genotype (OR = 1.03, 95% CI = 0.97–1.08) (Fig. 3C). Cumulative analysis confirmed the above results (Figure 4).

### Tests of Heterogeneity and Subgroup Analysis

Analysis of the following genetic models identified significant heterogeneities: B2B2 versus B1B1 (*P* = 0.04), B1B2 versus B1B1 (*P* = 0.004), and dominant (*P* = 0.001). Therefore, we used a random effects model in these analyses. Furthermore, we performed subgroup analysis according to ethnicity and found significantly decreased MI risk in the analysis of the B2B2 versus B1B1 (OR = 0.79, 95% CI = 0.68–0.92, *P* = 0.002) and recessive genetic models (OR = 0.84, 95% CI = 0.78–0.91, *P* < 0.001) among white populations. However, there was no significant association between the TaqIB polymorphism and MI risk among Asian populations. Table 2 lists the results in detail.

**TABLE 2:**
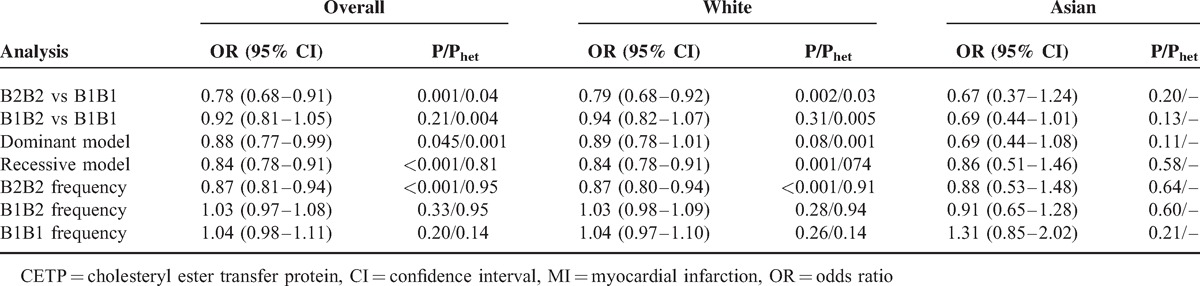
Summary ORs and 95% CIs of CETP TaqIB Polymorphism and MI

### Sensitivity Analysis

**FIGURE 5:**
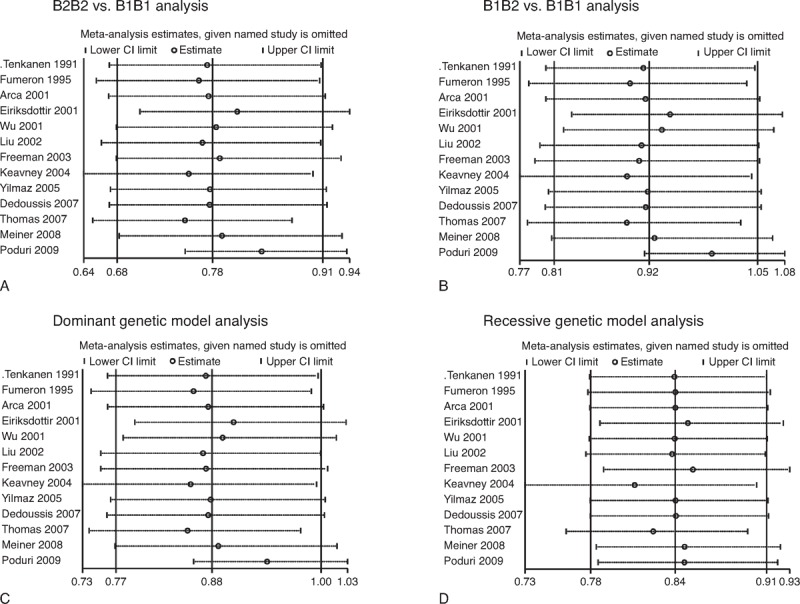
Influence analysis of *CETP* TaqIB polymorphism and MI risk. (A) B2B2 vs B1B1 analysis. (B) B1B2 vs B1B1 analysis. (C) Dominant genetic model analysis. (D) Recessive genetic model analysis. CI = confidence interval, MI = myocardial infarction, OR = odds ratio.

We conducted sensitivity analysis to assess the influence of each study on the pooled ORs by sequential omission of individual studies. The results indicated that the individual studies did not affect the pooled ORs in the analysis of the 4 genetic models (Figure 5).

### Publication Bias

**FIGURE 6:**
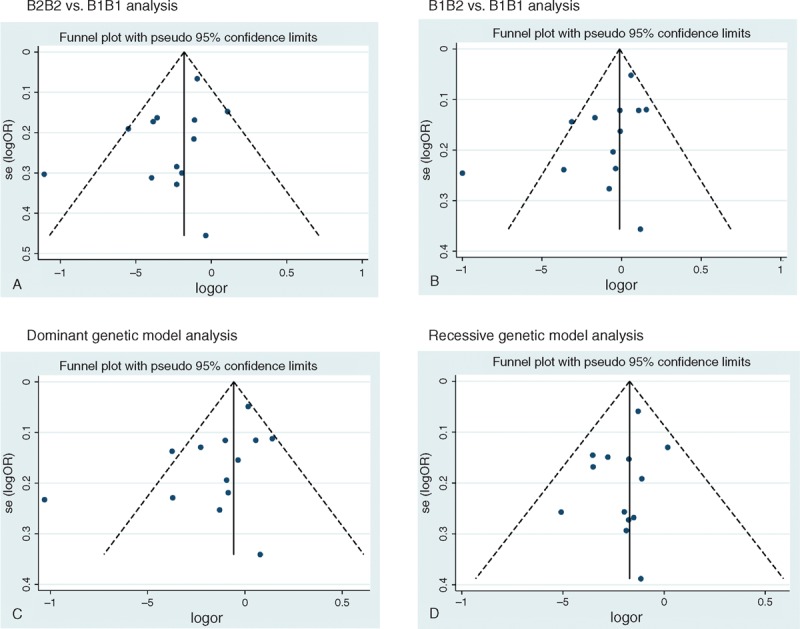
Funnel plot of *CETP* TaqIB polymorphism and myocardial infarction risk for publication bias. (A) B2B2 vs B1B1 analysis. (B) B1B2 vs B1B1 analysis. (C) Dominant genetic model analysis. (D) Recessive genetic model analysis.

Publication bias was examined using funnel plots, and no obvious asymmetry was observed in the analysis of the 4 genetic models (Figure 6). Egger test also did not reveal any evidence of publication bias; however, Begg test suggested potential publication bias in the B1B2 versua B1B1 analysis (*P* = 0.04).

## DISCUSSION

Our meta-analysis of the *CETP* TaqIB polymorphism and risk of MI included 13 studies involving a total of 8733 MI cases and 8573 controls. There was decreased MI risk in the analysis of the B2B2 versus B1B1, dominant, and recessive genetic models, which was confirmed by cumulative analysis. Moreover, the frequency of the B2B2 genotype was lower in MI cases. These results strongly suggest that the B2B2 genotype of the TaqIB polymorphism can serve as an independent protective factor against the development of MI. Given the large sample size in this meta-analysis, we believe that our results are robust and reliable.

It is well known that HDL could mediate reverse transport of cholesterol and decrease plasma cholesterol concentration.^32^ Therefore, it is accepted that high plasma HDL concentrations are associated with reduced risk of MI.^33^ CETP is a hydrophobic glycoprotein and catalyzes the transfer of cholesteryl esters from HDL to other lipoproteins, playing a pivotal role in HDL reverse transport.^34^ Higher CETP concentrations and/or activity decrease plasma HDL concentrations and increase LDL and VLDL fractions, which may contribute to increased risk of CHD, including MI.^35^ The TaqIB polymorphism of the *CETP* gene is a silent base change affecting nucleotide 277 in intron 1,^36^ and its role has been well studied. It has been reported that, compared with the B1 allele, the B2 allele of the TaqIB polymorphism is associated with larger HDL particles, higher plasma HDL-C, and lower plasma CETP activity^37^; there is also evidence that the B2B2 genotype increases HDL levels and that the B1 allele is closely associated with low HDL levels.^38^

Building on these findings, other studies have found that the B1 allele may increase the risk of CHD and MI or that the B2 allele may decrease this risk. The meta-analysis by Li et al^17^ suggested a positive association between the B1 allele of the TaqIB polymorphism and CAD susceptibility in the Han Chinese population. The study by Dedoussis et al^27^ found a protective effect of the B2B2 genotype against the development of acute coronary syndrome. Freeman et al^24^ found that individuals carrying the B2B2 genotype had 30% reduced risk of a cardiovascular event compared with B1B1 homozygotes; however, other studies failed to detect the protective effect of the B2B2 genotype in the selected population. Supporting the different functions of the B2 and B1 alleles in CETP activity and plasma HDL concentration, our meta-analysis results suggest that the B2B2 genotype plays a protective role against the development of MI. However, this protective role was found only among white populations, and not Asian populations. As there was only 1 study on Asian populations, more studies are warranted to explore this issue.

Although the pooled results of this meta-analysis are suggestive, it is necessary to mention its limitations. First, the included studies were not restricted to case–control studies; we also included observational studies and cohort studies in the pooled analysis. Therefore, the results may be biased. Second, there was significant heterogeneity in some of the pooled analysis, which may have affected the meta-analysis results even though we adopted the random effects model. Third, the genotype distributions in the controls in the studies by Wu et al,^22^ Keavney et al,^25^ and Thomas et al^28^ were not in HWE; therefore, the results may be biased. Lastly, the meta-analysis results were based on unadjusted estimates because most of the studies did not contain these results. In fact, environmental factors such as smoking and alcohol consumption could have modulated the effect of the polymorphism^19,39,40^; therefore, further studies based on these factors are warranted.

In conclusion, this comprehensive study evaluated all data currently available on the TaqIB polymorphism and MI risk. Our meta-analysis suggests that the B2B2 genotype of the TaqIB polymorphism is a protective factor against the development of MI, especially among white populations, which could be due to the association between the B2 allele of the TaqIB polymorphism and larger HDL particles, higher plasma HDL-C, and lower plasma CETP activity as compared with the B1 allele.
